# Knowledge and adherence to the Mediterranean diet in individuals practicing regular amatorial physical activity: a cross-sectional study conducted in the Metropolitan Area of Palermo, Italy

**DOI:** 10.3389/fpubh.2023.1204155

**Published:** 2023-06-23

**Authors:** Claudio Costantino, Alessandra Casuccio, Miriam Belluzzo, Francesco Balsamo, Nicole Bonaccorso, Alessandro Carubia, Luciano D’Azzo, Luisa Gattuso, Maria Chiara Lo Porto, Martina Sciortino, Tania Vitello, Garden Tabacchi, Francesco Vitale, Walter Mazzucco

**Affiliations:** ^1^Department of Health Promotion, Maternal and Infant Care, Internal Medicine and Medical Specialties (PROMISE) “G. D’Alessandro”, University of Palermo, Palermo, Italy; ^2^Emergency Medicine Department, University of Milano Bicocca, Monza, Italy; ^3^Department of Psychology, Educational Science and Human Movement, Sport and Exercise Sciences Research Unit, University of Palermo, Palermo, Italy

**Keywords:** Mediterranean diet, adherence, knowledge, amatorial physical activity, cross-sectional study

## Abstract

**Introduction:**

Mediterranean Diet (MD) is a universal model of nutrition that prevents several metabolic, cardiovascular, and oncological diseases. Main objective of the present study was to analyze adherence and knowledge regarding MD principles in a sample of individuals practicing amatorial sports from the Metropolitan Area of Palermo.

**Methods:**

A cross-sectional study was conducted in 10 Sports Centers, between October 2020 and September 2021, through a previously validated anonymous questionnaire structured in five sections including 74 items.

**Results:**

Overall, 337 subjects answered to the questionnaire. Based on the multivariable analysis conducted, a higher knowledge score (KS) on MD principles was observed among individuals daily consuming vegetables (OR: 3.32; CI95%: 1.82–6.02) and in the ones more adherent to MD principles (OR: 10.15; CI95%:5.47–18.85). More in depth, using MEDAS score to analyze the adherence to MD, a significant lower adherence was observed among overweight/obese (OR: 0.57; CI95%:0.33–0.99) and among employed subjects (OR: 0.52; IC95%: 0.28–0.98); while, a higher adherence was highlighted among daily consumers of vegetables (OR: 2.52; CI95%:1.52–4.17), daily consumers of fruit (OR: 1.77; CI95%:1.08–2.90), and in individuals that have daily breakfast (OR: 4.29; CI95%:1.15–15.96).

**Discussion:**

In accordance with the WHO Europe Gaining Health Campaign, Public Health Authorities should simplify accessibility to healthy food among general population, promoting principles and accessibility to MD.

## 1. Introduction

The Mediterranean diet (MD) represents a valid and certified model of nutrition for a proper lifestyle (1). Although the term “diet” is often associated to the lower amount of food intake with a weight loss goal, it is a correct amount and balance of nutrients that can really benefit health (1). In 2010, MD officially became a UNESCO World Intangible transnational Heritage, having recognized its importance: it is a lifestyle that emphasizes the values of hospitality and dialog and respects diversity, bringing together people of all ages, conditions and social classes, and unifying values that bring with them physical, psy-chological, and nutritional well-being for the individual (2).

Physical activity and active lifestyle are considered key components of the MD because they help in preventing many sedentary related diseases, such as obesity, hypertension, and atherosclerosis (3–5). Regular exercise reduces mortality from various non-communicable diseases (cardiovascular events, hypertension, stroke, metabolic diseases, type 2 diabetes, colon and breast cancer, depression, and falls associated with aging), improves endothelial function, and it can also ease autonomic dysfunction associated with aging (6–9). The amount of energy consumed depends on the intensity of the activity and the frequency of training, and varies according to the individuals’ age, gender, and body mass. Balance in the quantity and quality of food is essential for physical and mental development, while imbalance can cause illness and even death (10).

Mediterranean Diet has been identified as an important prevention factor for obesity and related disorders (11). MD prevents many diseases because it is associated with better cardio-vascular health outcomes, including clinically meaningful reductions in rates of coronary heart disease, ischemic stroke, and total cardiovascular disease: several studies have shown a high correlation between the average percentage of dietary energy from saturated fatty acids and the 10-year incidence and death rate from coronary heart disease 15-year follow-up confirmed these general relationships between coronary mortality rates and cohort mean characteristics of serum cholesterol, blood pressure, and the percentage of total energy from saturated fatty acids (12–15).

The PREDIMED study demonstrated a consistent reduction in major cardiovascular events, with a down-regulation of cellular and humoral inflammatory pathways linked to atherosclerosis, in subjects who adhered to the MD supplemented with extra virgin olive oil or walnuts compared to the control group following a low-fat diet (with a relative difference of 30% and an absolute difference of 1.7–2.1%). The fact that these beneficial effects were observed in older adults at high cardiovascular risk suggests that it is never too late to change eating habits to improve health status and that the MD can play a key role in preventing cardiovascular disease at any time (16).

Walnut consumption has been shown to be a protective factor against diabetes, along with reducing meat and dairy intake and increasing fiber intake, as observed in the MD (17, 18). Furthermore, adherence to MD during pregnancy has been associated with a lower risk of neural tube defects (19), premature births (20, 21) and fetal growth problems (22), as well as a reduction in waist circumference in pre-school children (23).

The PASSI surveillance system, a survey that collects information about the Italian adult population on lifestyles and on the behavioral risk factors associated with the incidence of chronic non-communicable diseases, provides a framework with many contrasts (24). Trends concerning overweight and obesity, physical activity and sedentariness, fruit and vegetable consumption, diabetes, and cardiovascular risk have been worsening for years according to a North–South gradient. Oddly enough, the worst results were observed in the southern regions, which, as they face the Mediterranean Sea, are rightfully part of that area historically considered as the cradle of MD: most likely, the reduced observance of MD and the poor awareness of the benefits it can bring, of which the above-mentioned factors may be a proxy, are to be found in social, economic and cultural reasons that characterize the southern regions and distinguish them from the rest of the Italian areas (24).

The aim of this study was to analyze knowledge and adherence to MD principles in an everyday-life setting, in a sample of individuals practicing amatorial sports in the Metropolitan Area of Palermo. The choice of this target was linked to the fact that practicing regular physical activity is one of the “pillars” of the MD and was amply demonstrated and if integrated with a correct diet can lead to a general well-being (25).

## 2. Materials and methods

### 2.1. Study design and participants

A cross-sectional study was conducted, from October 2021 to February 2022, through the administration of a self-administered digital questionnaire on a sample of individuals aged between 18 and over 65 years who practiced weekly recreational / amateur sports activities on a voluntary basis. More specifically, the survey was aimed at analyzing knowledge, attitudes, and adherence to MD in individuals recruited in six of the major sports centers in the province of Palermo (fitness centers, tennis and padel centers, and swimming pools). Based on the 34.9% of Sicilian adults that regularly practice physical activity in 2020–2021 (prevalence) (24) with a 95% desired level of significance the minimum study population needed to enroll to have statistical significance of the study sample was 315 subjects.

The administration of the questionnaire took place through dedicated links or QR Code created on the Google Documents® platform (26). The questionnaire was anonymous and in Italian. Purposes of the study, methods of treatment, conservation and protection of personal data were explained, and an informed consent form was signed and collected.

The reliability and validity of the questionnaires were evaluated in a preliminary pilot testing study conducted among 65 adults. In this study, Cronbach’s alpha was calculated and corresponded to 0.854, with an adequate reliability of the test.

This study was approved in September 2021 by the Ethics Committee Palermo 1 (Session no. 7 of 2021).

### 2.2. Questionnaire description

The questionnaire consisted of *74 items* distributed in five sections with the purpose of investigating:

1. Socio-demographic aspects: age, gender, anthropometric parameters (weight and height), residence, educational level, family members, work location, presence of chronic diseases, and type of disease.
2. Physical activity-related habits (how often and for how long physical activity is practiced, number of sports played, how COVID-19 has changed habits).
3. Eating/drinking habits (cooking routine, place where usually have a meal, knowledge and attitudes regarding MD, willingness to change eating habits, role of physicians and specialists in promoting MD).
4. Adherence to the MD, through a score obtained from the responses to 14 questions of the Mediterranean Diet Adherence Screener (MEDAS), validated and used in the PREDIMED study (15).
5. Knowledge and false beliefs about MD, through the Knowledge Score (KS) obtained from the true-false responses to the 12 included questions.

For the purposes of the present study, the MEDAS and KS scores are further described.

### 2.3. MEDAS score

The MEDAS Score, to which the fourth section is dedicated, is the score chosen to understand the adherence of individuals to the MD. Specifically, the MEDAS Score was previously validated and used in the PREDIMED study (15). In this section of the questionnaire, each participant was asked to answer *14 questions* about daily food habits with the aim of understanding the adherence of everyone. One point was awarded for using olive oil as the main source of fat for cooking, preferring white meat over red meat, or for consuming: two or more tablespoons (1 tablespoon = 13.5 g) of olive oil/day (including that used for frying, salads, meals consumed from home, etc.); two or more servings of vegetables/day; three or more fruit/day; <2 servings of red meat or sausages/day; <1 portion of animal fat/day; <1 sweetened drink/day; three or more portions of legumes/week; three or more portions of fish/week; <3 sweets, biscuits or pastry products/week; three or more servings of dried fruit/week; seven or more portions of wine, beer of 33 cL/week; and consumption of white meats preferably over red ones, if the condition is not met, zero points have been recorded for the category. The total MEDAS score ranges from 0 to 14, with a higher score indicating better adherence to MD. MEDAS score ≥ 7 represents a modest agreement and a score ≥ 9 represents strong agreement with the healthy diet model (15).

### 2.4. Knowledge score

The KNOWLEDGE Score, to which the fifth section is dedicated, is the score used to understand the degree to which individuals are aware of MD, already validated and used in the PREDIMED study (15). In this section of the questionnaire, each participant was asked to answer 13 questions about daily alimentary habits, with the aim of understanding each person’s knowledge. The subjects answered True or False to the following questions:

- physical activity is a basic foundation of the food pyramid;
- red meat, according to the MD, should be consumed monthly;
- it is advisable to maintain a moderate consumption of cold cuts, with a maximum of one portion (or if possible, even less) per week;
- peanuts are legumes;
- extra virgin olive oil (EVO) contains vitamins, antioxidants and has a beneficial effect on health;
- worldwide, more than 400 million people are affected by type 2 diabetes mellitus;
- white meats can be eaten weekly;
- pork and kid are white meats;
- for fish is indicated the same consumption as white meat;
- in a diet to lose weight you need to make five well balanced meals a day (three main and two minor);
- it has been scientifically proven that a good adherence to the MD prevents the onset of chronic-degenerative diseases (cardiovascular diseases, tumors, etc.); and
- food control rules are the same in all countries.

From the answers obtained a knowledge score ≤ 9 was considered related to a low level of knowledge, while a score ≥ 9 to a high level of knowledge.

### 2.5. Statistical analysis

Data obtained were collected in a Microsoft® Excel database, which was automatically filled by the Google® Modules online questionnaire administration system. Data were analyzed using STATA14® software (StataCorp. 2015. College Station, TX, United States: StataCorp LP). Absolute and relative frequencies were calculated for categorical variables. Differences in qualitative variables were analyzed using chi-square tests (or Fisher’s exact test, when appropriate). In the univariate analysis, the factors were included in a multivariate backward stepwise logistic regression model. In addition, all the variables with a value of *p* ≤ 0.20 associated to adherence to the MD at MEDAS Score and KNOWLEDGE Score were selected in the multivariate model, to guarantee a more conservative approach. Crude odds ratios (ORs) and adjusted ORs (adj-ORs), with their 95% CIs, were calculated. The level of significance chosen was a value of *p* < 0.05 (two tailed).

## 3. Results

The total number of individuals adhering to the survey was 337, with a response rate (RR) of 87%. As reported in Table 1, respondents were predominantly male (*n* = 186; 55.2%) and mostly with ages under 40 (57.3%). About the educational level, 64.7% of respondents had a degree equal to or greater than a university degree, followed by 35.3% of participants with a high school license; most of the interviewed were employed (*n* = 279; 72.8%). Furthermore, 27% of respondents were Overweight/Obese as reported in Table 1.

**Table 1:** Socio-demographic characteristics of the individuals practicing amatorial sports recruited in the survey (*n* = 337).

	*n* (%)
Gender	
Male	186 (55.2)
Female	151 (44.8)
Age	
≥40 years old	144 (42.7)
<40 years old	193 (57.3)
Weight status	
Underweight/normal weight	246 (73)
Overweight/Obese	91 (27)
Educational level	
Graduate/Post graduate	218 (64.7)
High school license	119 (35.3)
Occupation	
Employed	279 (72.8)
Unemployed	58 (17.2)

With regard to lifestyles, almost all the sample practiced physical activity weekly (97.3%) and had daily breakfast (93.2%); 73.6% of respondents habitually consumed alcohol, while only 42.2% of them adhered to MD. Specifically, there was evidence of daily consumption of fruits and vegetables by 36.2 and 52.2% of the sample, respectively (Table 2).

**Table 2:** Lifestyles reported by the individuals practicing amatorial sports recruited in the survey (*n* = 337).

	Yes	No
Weekly physical activity	328 (97.3%)	
≤3 h/week	56 (16.93%)	
>3 h/week	281 (85.67%)	
Daily alcohol consumption	248 (73.6%)	89 (26.4%)
Daily breakfast	314 (93.2%)	23 (6.8%)
Daily vegetable consumption (≥2 servings)	176 (52.2%)	161 (47.8%)
Daily fruit consumption (≥3 fruits)	122 (36.2%)	215 (63.8%)
Weekly read meat (included processed meat) (≥2 servings)	179 (53.10%)	158 (46.88%)
	High (≥ 9)	Low (< 9)
MD* knowledge score	195 (57.8%)	142 (42.2%)
MD* adherence score	142 (42.2%)	195 (57.8%)

^*^MD: Mediterranean Diet.

In Table 3, the univariate and multivariable analyses of factors associated with MD knowledge score was showed. In the multivariable model, factors significantly associated with better knowledge of the MD were individuals that daily consume vegetables (OR: 3.32; 95%CI: 1.82–6.02) and subjects that showed at minimum a modest adherence to MD (Medas Score ≥ 7; OR: 10.15; 95%CI: 5.47–18.85).

**Table 3:** Factors associated with MD^*^ knowledge score in the sample of individuals practicing amatorial sports recruited in the survey (*n* = 337).

	Crude OR^**^ (95% CI)	*p* value	Adj-OR^**^ (95% CI)	*p* value
Age				
< 40 years old	*Ref.*	0.72		
≥40 years old	0.92 (0.59–1.43)			
Gender				
Male	*Ref*	**<0.001**	*Ref*	*0.08*
Female	2.23 (1.42–3.50)		1.73 (0.93–3.21)	
Weekly physical activity				
≤3 h/week	*Ref*	**<0.20**	*Ref*	*0.49*
>3 h/week	4.28 (0.85–21.57)		2.02 (0.26–15.22)	
MEDAS score				
Modest (≥ 7)	*Ref*	**<0.001**	*Ref*	*<0.001*
Low (<7)	11.9 (6.72–21.19)		10.15 (5.47–18.85)	
Occupation				
Unemployed	*Ref*	0.49		
Employed	0.81 (0.45–1.46)			
Weight status				
Underweight/Normal weight	*Ref*	**<0.05**	*Ref*	0.75
Overweight/Obese	0.48 (0.29–0.78)		0.90 (0.47–1.7)	
Daily alcohol consumption				
No	*Ref*	0.6		
Yes	1.13 (0.7–1.9)			
Educational level				
High school	*Ref*	**<0.20**	*Ref*	*0.83*
Graduation/post-graduation	1.74 (1.11–2.75)		0.94 (0.52–1.68)	
Daily vegetable consumption (servings)				
<2	*Ref*	**<0.001**	*Ref*	*<0.001*
≥2	5.06 (3.15–8.09)		3.32 (1.82–6.02)	
Daily fruit consumption (number)				
< 3	*Ref*	**<0.05**	*Ref*	*0.72*
≥3	1.69 (1.06–2.69)		0.88 (0.48–1.62)	
Weekly red meat, included processed meat (servings)				
<2	*Ref*	**<0.05**	*Ref*	0.94
≥2	0.63 (0.40–0.97)		0.97 (0.55–1.71)	
Daily breakfast				
No	*Ref*	**<0.05**	*Ref*	0.25
Yes	3.19(1.26–8.06)		1.87 (0.64–5.50)	

^*^MD: Mediterranean Diet; ^**^OR: Odds Ratio; MD* Knowledge Score ≥ 9 vs. MD* Knowledge Score < 9 (KS). Statistically significant values have been shown in bold.

Table 4 reported the results of the univariate and multivariable analyses of factors associated with MD adherence explored by the MEDAS score. In the multivariable model, the following associations were highlighted: a lower adherence (MEDAS score) to MD among overweight/obese individuals (OR: 0.57; CI95%:0.33–0.99), and among employed subjects (OR: 0.52; IC95%: 0.28–0.98); a higher adherence to MD in individuals daily consuming vegetables (OR: 2.52; CI95%:1.52–4.17), daily consuming fruit (OR: 1.77; CI95%:1.08–2.90), and having daily breakfast (OR: 4.29; CI95%:1.15–15.96).

**Table 4:** Factors associated with MD* adherence explored by the MEDAS score in the sample of individuals practicing amatorial sports recruited in the survey (*n* = 337).

	Crude OR^**^ (95% CI)	*p* value	Adj-OR^**^ (95% CI)	*p* value
Age				
< 40 years old	*Ref.*	0.94		
≥40 years old	1.02 (0.65–1.57)			
Gender				
Male	*Ref*	0.23		
Female	1.30 (0.84–2.01)			
Weekly physical activity				
No	*Ref*	0.09	*Ref*	0.38
Yes	6.03 (0.74–48.7)		2.71 (0.29–25.67)	
Occupation				
Unemployed	*Ref*	0.06	*Ref*	**<0.05**
Employed	0.57 (0.32–1.02)		0.52 (0.28–0.98)	
Weight status				
Underweight/Normal weight	*Ref*	**<0.05**	*Ref*	**<0.05**
Overweight/Obese	0.48 (0.28–0.80)		0.57 (0.33–0.99)	
Alcohol consumption				
No	*Ref*	**<0.20**	*Ref*	0.18
Yes	1.41(0.86–2.34)		0.45 (0.84–2.50)	
Educational level				
High school/	*Ref*	**<0.05**	*Ref*	0.41
Graduation / post-graduation	1.94(1.21–3.1)		1.24 (0.748–2.09)	
Daily vegetable consumption (servings)				
<2	*Ref*	**<0.001**	*Ref*	**<0.001**
≥2	3.16 (2.01–4.99)		2.52 (1.52–4.17)	
Daily fruit consumption (number)				
<3	*Ref*	**<0.001**	*Ref*	**<0.05**
≥3	2.27 (1.44–3.58)		1.77 (1.08–2.90)	
Weekly red meat, included processed meat (servings)				
<2	*Ref*	**<0.20**	*Ref*	0.79
≥2	1.41(0.86–2.34)		1.07 (0.65–1.74)	
Daily breakfast				
No	*Ref*	**<0.20**	*Ref*	**<0.05**
Yes	5.29 (1.54–18.18)		4.29 (1.15–15.96)	

^*^MD: Mediterranean Diet.

^**^OR: Odds Ratio.

MD* Medas Score ≥ 9 vs Medas Score ≤ 8 to MD* (MEDAS Score).

Statistically significant values have been shown in bold.

## 4. Discussion

This cross-sectional study was focused on the importance of the MD, included by UNESCO in the list of intangible cultural heritage in 2010 (2). We have tried to investigate the level of knowledge and adherence to this diet, in an everyday-life setting, through a questionnaire administered to a sample of individuals practicing amatorial sports in the Metropolitan Area of Palermo. The results of our study showed that even in such a selected population there are still few individuals who adhere to the cornerstones of MD (42.2%), probably because in modern and industrialized populations there is a tendency to eat unhealthy and faster meals (27).

Mediterranean Diet is a nutrition model based on the balanced intake of micro and macronutrients which, in synergy with regular physical activity, can positively influence the well-being of everyone by preventing metabolic, cardiovascular, and cancer diseases, and it promotes longevity as well.

In a study conducted on a sample of school-age individuals, it was seen that lessons aimed at raising awareness of MD principles lead to increased consumption of fruits and vegetables, resulting in increased adherence to the MD itself (28). Indeed, a diet rich in fruits, vegetables and a regular breakfast affect the nutritional status of people and their health, thus increasing the degree of adherence to the MD with consequent reduction in the incidence of cardiovascular disease, cancer, and other serious diseases (29).

The Mediterranean Diet is not only a great dietary guideline for healthy individuals engaging in sports (30–40) but is also particularly beneficial for those with health conditions, taking into account necessary adjustments and precautions. The rise of chronic degenerative diseases can be attributed to poor diet and decreased physical activity, which are consequences of technological advancements, impacting not just industrialized countries but also in emerging ones (30).

A study on Cypriot children having regular breakfast showed greater adherence to the MD (41). Also, who consumes vegetables and fruit daily obtained a higher MEDAS score, and this was in line with a study conducted on Spanish university students, stating that high level of adherence to the MD is associated with consumptions of more than two servings of vegetables/day and three or more fruits/day (42). Weight overload was associated with body dissatisfaction and a low score on the Mediterranean diet adherence test (43); in other studies, it has been seen that even in pediatric age being overweight-obese involves a reduction in the degree of adherence to MD (44).

As highlighted in the results, unemployed individuals showed a higher adherence to the MD, probably because those who do not have a job, having more time available than those who have stable jobs, are able of choosing the best foods calmly and, therefore, preparing healthier meals to eat. The time factor emerges also from studies that have evaluated adherence to the MD during the various lockdowns caused by the COVID-19 pandemic (45, 46), highlighting how having more spare time, due to the confinement at home, allowed people to prepare healthier meals and thus follow a healthier dietary regimen.

A study conducted by Pfeifer et al. (46), reported an increase in cooking frequency during confinement associated with increased consumption of vegetables, legumes, fish, and seafood. The increased adherence to MD by those who know it best can most likely be attributed to the fact that by informing themselves about this dietary regimen, even independently, they can appreciate its health benefits and thus consider it as a viable alternative to any other diet.

These results are confirmed by other researches that investigated the association between the level of adherence and the level of knowledge on the MD, such as the one conducted by Aureli et al. (47), who observed a close correlation between adherence and knowledge of MD in a sample of 2,869 adults, with adherence increasing as knowledge increased and vice versa, in a context where, with the passage of time due to globalization and ever faster rhythms, adherence to the MD progressively decreases. This should encourage health workers to commit to ensure more knowledge and appreciation by the general population to promote its positive effect on health.

Projects involving schools, for example, could give good results because students might end up involving parents by bringing home useful information to make it clear that eating well makes people feel good, thus creating a synergy that can lead to a clear improvement of the quality of the diet. The work conducted by Philippou et al. (48), for example, has highlighted how much the involvement of families can be useful to multiply the effects of an interactive educational intervention that involves not only adolescents but also those who are part of their life, thus creating a virtuous context. In accordance with the WHO Europe Gaining Health Campaign, Public Health Authorities should simplify accessibility of general population to foods that are healthy and that promote principles of MD (49).

Therefore, the fundamental objective of public health is to work as a team to reach all those subjects who for various reasons (work, education, age, etc.…) do not have the time or are unable to get information correctly; this could be realized by starting a whole series of initiatives involving various actors such as the local Food and Nutrition Hygiene Services, which have among their objectives the spreading of healthy lifestyles, with targeted projects in the workplace, schools, and universities (37, 50, 51); general practitioners, pediatricians, and other health professionals should be addressed as well, since, according to what emerged from the “PASSI Survey” (52), they could do much more to sensitize their patients to follow a healthier diet and to carry out regular physical activity.

This study has some limitations that need to be considered. The limited sample size might lead to a reduction in the study power. Secondly, the individuals of which the sample is formed practice recreational sport activities in sport centers and, therefore, might be keener than those who do not practice any sporting activity to learn about other diets, including the MD, and this could be a selection bias; this could suggest to perform future studies to examine the comparison with a group of sedentary people and another group of elite/professional athletes. Lastly, another limitation could be the methodological approach based on the use of a self-administered questionnaire; although the instrument was previously validated, respondents may overestimate or underestimate some of the requested parameters, thus affecting the accuracy of the answers.

## 5. Conclusion

In an increasingly frantic world where people tend to eat quickly and without paying too much attention to what they ingest, an extra effort should be made to ensure MD principles becoming the preferred eating lifestyle by an ever-larger portion of the population.

It is essential that public health operators take charge of the situation by involving health professionals at every level, while designing and implementing at the same time initiatives targeted to schools and families, that allow to explain in a simple and clear way how a healthy diet, accompanied by regular physical activity, represents a good viaticum for an ever-higher quality of life.

## Data availability statement

The raw data supporting the conclusions of this article will be made available by the authors, ensuring the anonymity of the respondents to the questionnaire, without undue reservation.

## Ethics statement

The studies involving human participants were reviewed and approved by Palermo 1, Via del Vespro 129, 90,127, Palermo, Italy. The patients/participants provided their written informed consent to participate in this study.

## Author contributions

CC, LG, NB, FB, and MB: conceptualization. ACas, FB, GT, LG, and MS: methodology. TV, ACar, and LD’A: software. ACas, ACar, FB, NB, LG, GT, ML, LD’A, and MS: validation. ACas, TV, LG, ML, and FB: formal analysis. ACar, ML, TV, and LD’A: investigation. ACar, LD’A, MS, and GT: resources. ACas, NB, MS, TV, and ML: data curation. MB, CC, and WM: writing—original draft preparation. MB: writing, review, and editing. CC, MB, and FV: visualization. FV, WM, and CC: supervision. FV and WM: project administration. WM, GT, NB, and FV: funding acquisition. All authors contributed to the article and approved the submitted version.

## Conflict of interest

The authors declare that the research was conducted in the absence of any commercial or financial relationships that could be construed as a potential conflict of interest.

## Publisher’s note

All claims expressed in this article are solely those of the authors and do not necessarily represent those of their affiliated organizations, or those of the publisher, the editors and the reviewers. Any product that may be evaluated in this article, or claim that may be made by its manufacturer, is not guaranteed or endorsed by the publisher.
